# LAYING THE GROUNDWORK FOR BECOMING AN AGE-FRIENDLY UNIVERSITY: A MULTI-PHASE STUDY

**DOI:** 10.1093/geroni/igz038.305

**Published:** 2019-11-08

**Authors:** Melissa L Cannon, Renata Kerwood, Mandie Kondash, Samuel Rowley, Hannah Wehr

**Affiliations:** Western Oregon University, Monmouth, Oregon, United States

## Abstract

As evidenced by the growing Age-Friendly University (AFU) global network, institutions of higher education are increasingly seeking ways to enhance the experiences of older adults who use them for reasons such as lifelong learning, career development, and intergenerational engagement. This multi-phase study explored the barriers and facilitators for older adults accessing a public university in a small Oregon town. The first phase of the study involved survey data collection from 46 members of the local senior center adjacent to the university campus. For the second phase, researchers recruited a sample from the survey respondents and used snowball sampling to conduct 12 interviews with senior center members, past and current senior center directors, and key contacts among university staff. The third phase of data collection paired student researchers with older adult research participants (N=14) in participatory action research to capture the unique perspectives of the research participants visiting the university campus using photovoice and a follow-up focus group. Quantitative data were analyzed using SPSS while qualitative data were analyzed using team coding and intensive group discussion to develop categories and themes. Findings revealed various opportunities to increase age-friendliness according to the principles outlined by the AFU initiative, such as developing a lifelong learning center on campus, strengthening the university-senior center partnership, and removing accessibility barriers in order to make the university campus friendlier for people of all ages and abilities. These findings are being used in a proposal to join the AFU network and to shape the university’s AFU vision.

